# Supplementary material for: Identification of photoperiod-induced specific miRNAs in the adrenal glands of Sunite sheep (Ovis aries)
Source: Front Vet Sci. 2022 Jul 22;9:888207. doi: 10.3389/fvets.2022.888207 (PMC9354845; doi:10.3389/fvets.2022.888207)
Supplement: Supplementary file 1 [file Data_Sheet_1.ZIP › Supplementary file/miRDeep2 file.zip!/miRDeep2 file/Know_Structure/SPLP42A3/oar-mir-411a.pdf]

miRBase precursor : oar-mir-411a
Total read count : 48911
oar-miR-411a-5p read count : 45241
oar-miR-411a-3p read count : 3666
remaining reads : 4

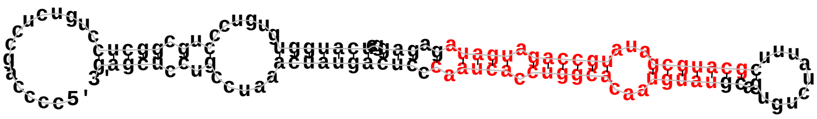

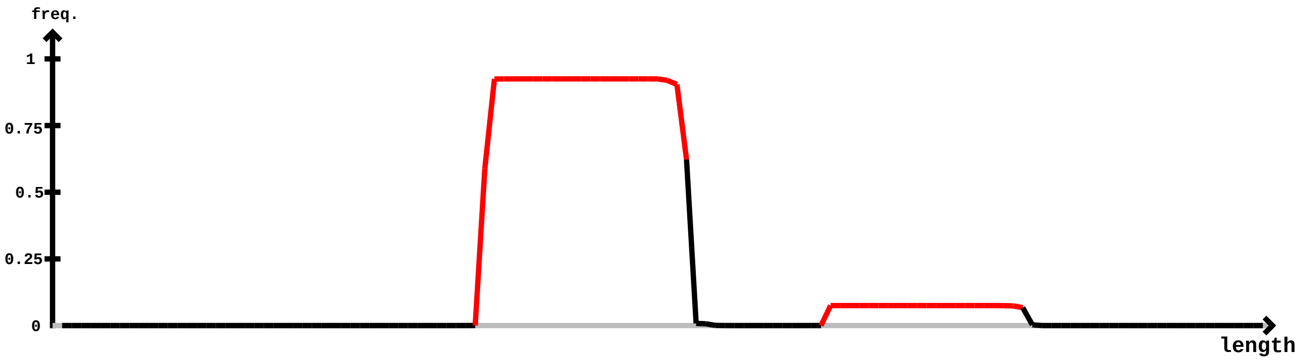

oar-miR-411a-3p

oar-miR-411a-5p

```
5'- ccccagccucuguccucggcgucccugugugguacucggagagauaguagaccguauagcguacgcuuuaucugugacguauguaacacgguccacuaacccucaguaucaaauccguccucgag  -3'   exp
    ..............(((((.(.(......(((((((..(((.(.((((.((((((...((((((((.........).)))))))...)))))).)))).).)))))))))).....).).)))))   reads     mm
    ......................cccugugugguacucggag....................................................................................   1         0
    ........................cugugugguacucggagaga.................................................................................   1         0
    .........................................agauaguagaccguauagcgu...............................................................   2         0
    .........................................agauaguagaccguauagcguac.............................................................   1         0
    ..........................................gauaguagaccguauagcguac.............................................................   3         0
    ..........................................gauaguagaccguauagcguacg............................................................   6         0
    ...........................................auaguagaccguauagcg................................................................   3         0
    ...........................................auaguagaccguauagcgu...............................................................   224       0
    ...........................................auaguagaccguauagcgua..............................................................   499       0
    ...........................................auaguagaccguauagcguac.............................................................   8578      0
    ...........................................auaguagaccguauagcguacg............................................................   19299     0
    ...........................................auaguagaccguauagcguacgc...........................................................   21        0
    ...........................................auaguagaccguauagcguacgcu..........................................................   101       0
    ...........................................auaguagaccguauagcguacgcuu.........................................................   2         0
    ............................................uaguagaccguauagcgu...............................................................   46        0
    ............................................uaguagaccguauagcgua..............................................................   239       0
    ............................................uaguagaccguauagcguac.............................................................   5194      0
    ............................................uaguagaccguauagcguacg............................................................   10745     0
    ............................................uaguagaccguauagcguacgc...........................................................   35        0
    ............................................uaguagaccguauagcguacgcu..........................................................   195       0
    ............................................uaguagaccguauagcguacgcuu.........................................................   24        0
    ............................................uaguagaccguauagcguacgcuuu........................................................   3         0
    .............................................aguagaccguauagcguac.............................................................   4         0
    .............................................aguagaccguauagcguacg............................................................   9         0
    .............................................aguagaccguauagcguacgcu..........................................................   7         0
    ..............................................guagaccguauagcguac.............................................................   1         0
    .......................................................auagcguacgcuuuaucugug.................................................   1         0
    .................................................................cuuuaucugugacguauguaacac....................................   1         0
    .............................................................................cguauguaacacgguccacuaa..........................   2         0
    ..............................................................................guauguaacacgguccacua...........................   1         0
    ...............................................................................uauguaacacgguccacu............................   14        0
    ...............................................................................uauguaacacgguccacua...........................   45        0
    ...............................................................................uauguaacacgguccacuaa..........................   308       0
    ...............................................................................uauguaacacgguccacuaac.........................   3156      0
```

```
                                                                               oar-miR-411a-3p
                                           oar-miR-411a-5p
ccccagccucuguccucggcgucccugugugguacucggagagauaguagaccguauagcguacgcuuuaucugugacguauguaacacgguccacuaacccucaguaucaaauccguccucgag

...............................................................................uauguaacacgguccacuaacc........................    137        0
................................................................................auguaacacgguccacuaac.........................    3          0
```
